# Large-Scale Production of Silver Nanoplates via Ultrasonic-Assisted Continuous-Flow Synthesis

**DOI:** 10.3390/nano15231770

**Published:** 2025-11-25

**Authors:** Xiangting Hu, Yixuan Yao, Fuqiang Yan, Jiahao Pan, Zhenda Lu

**Affiliations:** Department of Materials Science and Engineering, College of Engineering and Applied Sciences, Nanjing University, Nanjing 210093, China; huxtcn@163.com (X.H.); 522024340081@smail.nju.edu.cn (Y.Y.); 522025340077@smail.nju.edu.cn (F.Y.); jiahaopan1999@163.com (J.P.)

**Keywords:** continuous-flow synthesis, continuous-flow reaction device, silver nanoplates, large-scale synthesis

## Abstract

Silver nanoplates hold significant promise for advanced electronic materials, especially in low-temperature conductive silver pastes crucial for next-generation solar cells. However, their widespread practical application, like many nanomaterials, is currently limited by insufficient production capacity and inconsistent quality inherent in conventional batch synthesis methods. To overcome these critical challenges, we developed a novel ultrasound-assisted continuous-flow synthesis method for the scalable and high-yield production of silver nanoplates. This innovative approach effectively addresses common issues such as nanoparticle deposition and pipeline clogging by leveraging ultrasonic cavitation for enhanced mixing and stable flow. Through systematic optimization of synthetic parameters-including temperature, flow rate, and seed concentration-our continuous-flow reactor achieved mass production of pure silver nanoplates at a rate of 3.8 g/h. This scaled-up system is capable of producing hundreds of grams per day. The as-prepared nanoplates demonstrate excellent electrical performance, highlighting the method’s potential for industrial-scale manufacturing and significantly advancing the development of high-efficiency electronic devices.

## 1. Introduction

**Scheme 1:**
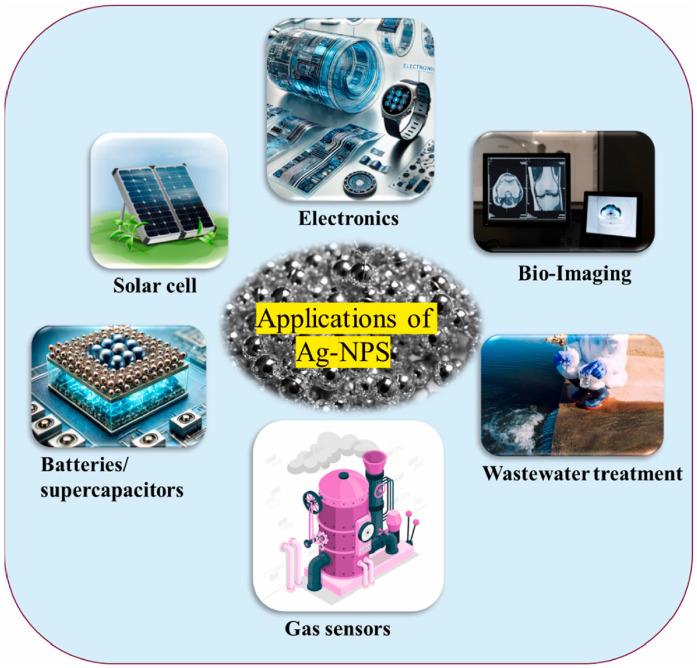
Schematic illustration of the widespread applications of silver nanoparticles [16].

Noble metal nanomaterials have attracted significant attention due to their exceptional optical [1,2,3], electrical [4,5], and catalytic [6] properties, leading to their broad application in diverse fields. Among these, silver nanomaterials are particularly prominent owing to their superior electrical and thermal conductivity, making them widely employed in diverse fields such as electronics [7], solar cells [8], and gas sensors [9], etc. (Scheme 1). Researchers have successfully synthesized silver nanomaterials with a variety of morphologies, including nanospheres [10], nanocubes [11], nanoplates [12,13], and nanowires [14,15], each possessing distinctive characteristics customized for particular functions.

Two-dimensional anisotropic silver nanoplates are of particular interest due to their distinctive electrical properties [17]. Compared to conventional spherical nanopowders, flake-like silver nanomaterials offer significant advantages. Their unique planar structure facilitates more effective surface and line contacts, substantially reducing contact resistance [18]. This sheet-like morphology also imparts superior electromagnetic shielding performance, improved slurry stability, and enhanced substrate adhesion. Furthermore, conductive pastes formulated with flake-shaped silver powders exhibit improved rheological properties [19]. These outstanding characteristics make silver nanoplates an important material in electronic devices and various functional applications [20,21].

Traditional methods for synthesizing silver nanoplates with controllable diameter-to-thickness ratios include mechanical ball milling [22], liquid-phase reduction [23], and photoinduced synthesis [24]. Seed-mediated growth stands out as a simple and reliable strategy for precisely controlling nanocrystal morphology and size, enabling the production of nanomaterials with desired properties [25,26,27]. This approach separates the nucleation and growth phases by introducing preformed seeds, allowing fine-tuning of nanocrystal morphology through adjustments to growth conditions [28]. However, conventional seed-mediated growth is typically conducted as a batch reaction in a closed reactor system. This batch nature presents considerable challenges for scalability and reproducibility, particularly when transitioning from laboratory-scale protocols to industrial production.

Flow chemistry, utilizing microreaction processes, offers a promising solution to these challenges. It enables precise control over reaction parameters, improves uniformity, and achieves higher throughput [29]. Microfluidic reactors have been widely adopted for synthesizing various oxides, salts, quantum dots, and other nanomaterials [30,31,32]. Notably, continuous-flow tubular microreactors have also been successfully employed for synthesizing narrowly dispersed silver nanoparticles [33]. A key advantage of microreactors is their ability to achieve target reaction temperatures in extremely short times (seconds or even milliseconds). Furthermore, unlike traditional batch reactors, flow reactors enable the continuous production of the target product once steady-state conditions are established [34].

Despite the successful application of flow microreaction technology in synthesizing various nanomaterials, its implementation has largely been confined to laboratory-scale production, with limited examples of industrial-scale amplification [35]. To our knowledge, reports on the large-scale production of silver nanoplates via flow chemistry remain scarce [36,37,38]. While flow microreactor technology theoretically offers a straightforward path to scaling up production by increasing reactor dimensions, this approach introduces new technical challenges. These include uneven distribution of heat and reactant concentrations along both the axial and radial directions of the reactor. Moreover, during continuous-flow synthesis, nanoparticles tend to aggregate and deposit within the tubing, leading to clogging and compromising production stability. Therefore, maintaining product quality and uniformity during scale-up remains a significant technical bottleneck.

To overcome these critical limitations, we propose a novel ultrasound-assisted continuous-flow method for the large-scale synthesis of silver nanoplates. While seed-mediated growth in batch systems has been extensively studied [39,40,41], its application in continuous-flow systems, especially for morphology-sensitive nanostructures, remains largely underexplored. Our approach integrates ultrasonic irradiation into the continuous-flow setup. This leverages cavitation effects to mitigate particle sedimentation and maintain homogeneous growth conditions. This not only prevents channel clogging but also significantly enhances mixing, directly addressing a critical bottleneck in the millimeter-scale continuous-flow synthesis of nanomaterials. We demonstrate the successful preparation of silver nanoplates using this ultrasound-assisted continuous-flow method, initially with a PTFE tubing reactor (3 mm inner diameter, 2 m length). Subsequently, we designed and constructed an upscaled synthesis system featuring a reactor with a larger inner diameter (5 mm), achieving a remarkable production capacity of 3.8 g/h of pure silver nanoplates.

## 2. Materials and Methods

### 2.1. Materials

Silver nitrate (AgNO_3_), trisodium citrate dihydrate (TSC, Na_3_C_6_H_5_O_7_·2H_2_O), L-ascorbic acid (AA, C_6_H_8_O_6_) and sodium sulfite (Na_2_SO_3_) were all purchased from Sinopharm Chemical Reagent Co., Ltd., Shanghai, China. Polyvinyl pyrrolidone (PVP) (Mw = 58,000), hydrogen peroxide (H_2_O_2_) and sodium Borohydride (NaBH_4_) were purchased from Aladdin Co., Ltd., Shanghai, China. All chemicals were used as received without further purification. Deionized water (DI water, 18.2 MΩ·cm) was obtained with ultra-pure water equipment. The organic carrier used for preparing conductive pastes was provided by Nantong Tiansheng New Energy Co., Ltd., Nantong, China.

### 2.2. Methods

The synthesis of Ag nanoplates via seeded growth in a single-channel continuous-flow reactor system. The channel used was PTFE pipes with inner diameters of 3 mm, placed in an ice water bath with ultrasound. The water bath temperature was maintained at 5 °C by continuously adding ice. Precursor A was a 100 mL aqueous solution of 0.06 M silver nitrate and precursor B was 100 mL aqueous solution of 0.1 M sodium sulfite. Precursor C consisted of 200 mL solution containing 0.05 M TSC, 0.05 M AA, 1.5 mM PVP, and a specified amount of seed solution. The seed solution was pre-synthesized via a previously reported chemical reduction approach [42].

The residence time of the reaction within the PTFE tube was regulated by modulating the propulsion speed of the syringe (i.e., different flow rates). The collected products were homogenized using a magnetic stirrer.

Based on the process parameters determined through laboratory experiments, an industrially scaled-up production process for Ag nanoplates was designed. While maintaining a maximum inner tube diameter of 5 mm, the production capacity was increased by extending the tube length.

The as-synthesized silver nanoplates dispersion from the continuous-flow reaction was subjected to a 12 h sedimentation. The collected sediment was redispersed and washed with deionized water and ethanol via centrifugation at 3500 rpm several times. The purified nanoplates were finally dried at 60 °C for 2 h. To prepare the conductive pastes, dried Ag nanoplates product was added into the commercially available organic carrier at a mass fraction of 51%, followed by dispersion of these pastes with a homogenizer (ALLSHENG BIOPREP-24, Hangzhou, China) to generate a homogeneous paste (at 3000 rpm, 10 cycles, 2 min per cycle). The patterns were screen printed onto PET films by using a screen printer. Then, the printed patterns were dried in an air-drying oven at 140 °C for 20 min. After cooling down, the electrical properties of the printed circuit were further tested by multimeter.

### 2.3. Characterization

The morphology of Ag nanoplates was studied with a field emission scanning electron microscope (FESEM, Ultra 55, Zeiss, Jena, Germany). The calculations of the distribution and size average were based on the nanoplates (>100) observed in the SEM image. The electrical resistance of conductive pastes doped with Ag nanoplates produced by this method was measured with a four-probe technique by using a digital multimeter (Keithley 2110, Cleveland, OH, USA). The thickness of the printed shape was measured using a laser microscope (Keyence VK series, Osaka, Japan).

## 3. Results and Discussion

### 3.1. Shape Control of Silver Nanoplates

The synthesis of silver nanoplates in this study largely follows a conventional seeded growth approach [43,44]. In our method, ascorbic acid (AA) serves as the reducing agent, facilitating the reduction of silver nitrate (AgNO_3_) to metallic silver nanoplates. To control the crystal growth process, trisodium citrate (TSC) is employed as a capping agent. TSC selectively attaches to the basal {111} facets of the silver seeds, effectively inhibiting vertical growth while promoting substantial growth along the lateral axis, thereby directing the formation of two-dimensional nanoplates. Additionally, poly(vinylpyrrolidone) (PVP) acts as a dispersant or surfactant, aiding in stabilizing the nanoparticles and preventing agglomeration. To precisely manage the nucleation process and prevent unwanted self-nucleation of silver, sodium sulfite is strategically utilized as a ligand for the Ag(I) salt. This coordination complex reduces the reduction potential of the Ag(I) salt, maintaining a low concentration of elemental Ag(0) even when the Ag(I) species concentration is high. This approach effectively minimizes unintended nucleation events by modulating the redox properties of the silver precursor.

### 3.2. Continuous-Flow System and Initial Characterization

**Figure 1:**
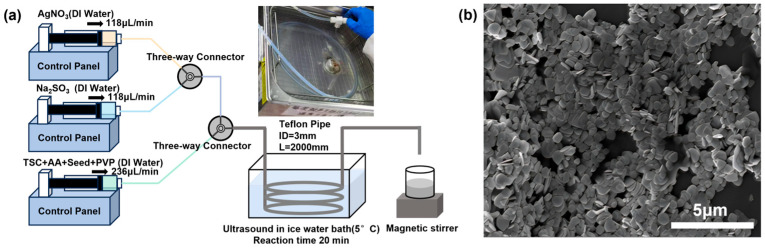
(**a**) Schematic illustration of the single-channel continuous-flow reactor system employed for the scalable synthesis of silver nanoplates. (**b**) Representative scanning electron microscopy (SEM) image showing the morphology of silver nanoplates produced via the continuous-flow method.

For continuous-flow synthesis, we constructed a single-channel reaction system (Figure 1a). This system involved three solutions: a silver nitrate solution (Solution A), a sodium sulfite solution (Solution B), and a mixed solution containing ascorbic acid, trisodium citrate, PVP, and the silver seed solution (Solution C). Solutions A and B were initially mixed, then combined with Solution C at a flow rate of 236 μL/min. The combined stream then flowed through a two-meter long Teflon pipe with a 3 mm inner diameter, which was immersed in a 5 °C ice-water bath. The reaction time was directly controlled by the flow rate. The final product dispersion was collected and continuously stirred for subsequent separation.

Representative scanning electron microscopy (SEM) images (Figure 1b) of the synthesized silver nanoplates confirmed the successful formation of two-dimensional, predominantly triangular and hexagonal nanoplates with sub-micron edge lengths. This continuous-flow reaction system demonstrated its capability for continuous production of high-quality silver nanoplates, yielding approximately 28.3 mL/h of dispersion, which corresponds to roughly 30 mg/h of Ag nanoplates.

### 3.3. The Role of Ultrasound Assistance

A significant hurdle in the continuous-flow synthesis of nanomaterials, particularly when scaling up pipe diameters beyond microfluidic dimensions, is the prevalent issue of particle agglomeration and non-uniform mass transfer. To address these challenges, our study introduces ultrasound-assisted technology integrated into the continuous-flow system.

**Figure 2:**
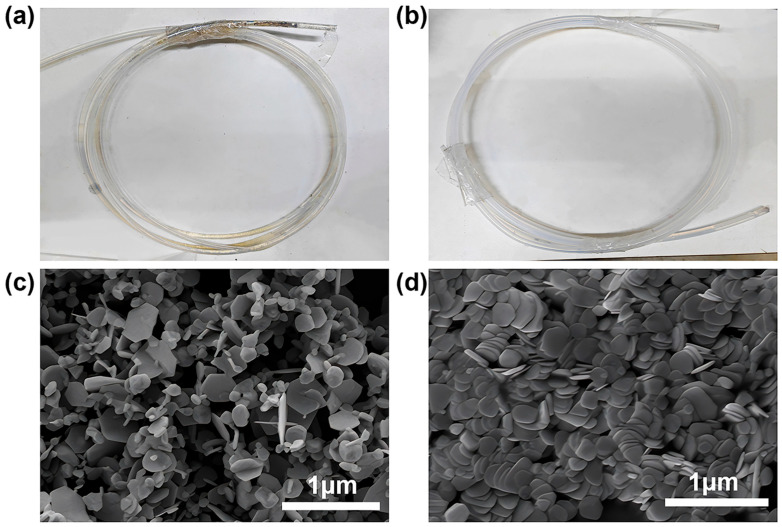
Impact of ultrasound on continuous-flow synthesis. (**a**,**b**) Images of PTFE tubes operating under non-ultrasonic and ultrasonic conditions, respectively, highlighting the prevention of clogging. (**c**,**d**) Corresponding SEM images of silver nanoplate products obtained without and with ultrasonic assistance, demonstrating improved dispersion and morphology.

As illustrated in Figure 2a,b, a clear contrast was observed between reaction channels with and without ultrasound treatment. Without ultrasonic assistance, significant clogging of the reaction channels occurred, indicative of severe particle aggregation and deposition. In contrast, the ultrasound-assisted conditions maintained largely unobstructed flow, demonstrating the effectiveness of this approach in preventing channel blockages. Further SEM analysis proved these findings: products obtained without ultrasonication displayed severe particle aggregation, whereas ultrasound-treated samples exhibited remarkable uniform dispersion with a parallel lamellar structure distribution.

This beneficial phenomenon is mainly attributed to ultrasonic cavitation effects, which induce localized turbulences and enhance mixing within the reaction microzones. Furthermore, the synergistic interplay between ultrasound and the continuous flow system disrupts the diffusion-limited layers that are characteristic of conventional continuous-flow reactions. This disruption significantly improves mass transfer uniformity throughout the reactor. Consequently, the incorporation of ultrasound assistance proved to be an essential requirement for the successful implementation of this experimental protocol, ensuring product quality and system stability.

### 3.4. Optimization of Synthesis Parameters in Continuous Flow

Continuous-flow systems offer distinct advantages for rapid and efficient screening of reaction conditions compared to traditional batch reactions. Therefore, we systematically investigated key synthesis parameters, including temperature, flow rate, and seed concentration, to optimize the production of silver nanoplates.

#### 3.4.1. Effect of Temperature

**Figure 3:**
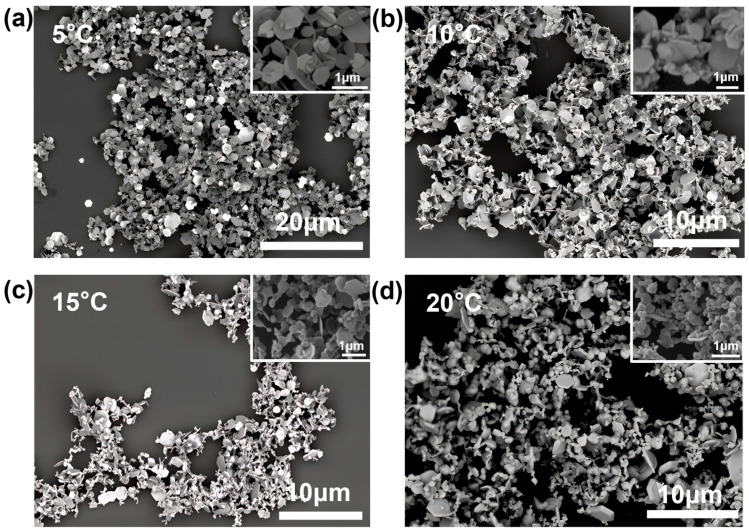
SEM images of silver nanoplates synthesized at various temperatures. The images correspond to reaction temperatures of (**a**) 5 °C, (**b**) 10 °C, (**c**) 15 °C, and (**d**) 20 °C. Insets are the corresponding SEM images at a higher magnification.

First, we evaluated the effect of temperature on product morphology while maintaining a reaction time of 45 min with other parameters unchanged. As depicted in Figure 3a, conducting the reaction at 5 °C yielded highly uniform silver nanoplates with minimal formation of undesired silver nanoparticles. However, as the temperature was incrementally increased to 10 °C and then to 15 °C, the uniformity of the silver nanoplates progressively deteriorated, accompanied by an increasing presence of silver nanoparticles (Figure 3b,c). At 20 °C (Figure 3d), nanoparticle formation became predominant, with only small amounts of nanoplates being produced.

These observations align with established principles of nanoparticle synthesis. Elevated temperatures accelerate the reduction rate of AgNO_3_. If this rate exceeds the nucleation threshold of elemental silver, it favors homogeneous nucleation of new particles in the bulk solution rather than their deposition onto pre-existing seeds. Therefore, to achieve well-defined silver nanoplates with high uniformity, maintaining the reaction temperature at 5 °C was important, as it ensured sufficiently slow reaction kinetics that favored anisotropic growth.

#### 3.4.2. Effect of Flow Rate (Residence Time)

Next, we systematically investigated the effect of flow rate (and consequently, the residence time of the reaction solution in the tubing) on the morphological control of silver nanoplates, targeting an average edge length of approximately 500 nm, using a 3 mm diameter tubular reactor.

**Figure 4:**
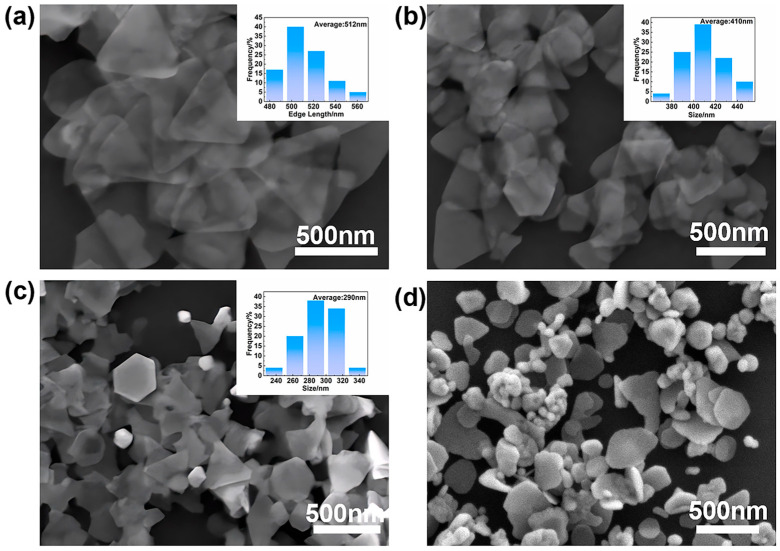
Effect of residence time (controlled by flow rate) on silver nanoplate growth. (**a**–**d**) Representative SEM images illustrating the morphology of silver nanoplates obtained at residence times of 30, 20, 10, and 5 min, respectively. Inset images show the corresponding size distributions of the silver nanoplates.

When the flow rate was controlled at 472 µL/min (residence time of 30 min), well-defined silver nanoplates with an average diameter of 500 nm were obtained (Figure 4a). Increasing the flow rate to 708 µL/min (residence time of 20 min) resulted in a slightly smaller average diameter of 410 nm, while still maintaining the uniform plate morphology (Figure 4b). Further elevation of the flow rate to 1416 µL/min (residence time of 10 min) yielded even smaller nanoplates (290 nm average diameter) but with noticeable edge defects and minor silver nanoparticle byproducts (Figure 4c). Finally, at a flow rate of 2832 µL/min (residence time of 5 min), the reaction time was insufficient, leading to the predominant formation of silver nanoparticles, with only fragmented and irregular small nanoplates observed (Figure 4d).

Mechanistic analysis revealed that longer residence times facilitated the oriented growth of silver nanoplates at constant seed concentrations, producing larger and more uniform nanostructures. However, an excessively long residence time (e.g., 30 min vs. 20 min) did not significantly improve product quality and, in some cases, tended to induce particle agglomeration. After balancing product quality with production efficiency, 708 µL/min (20 min residence time) was selected as the optimal flow rate. This condition simultaneously ensured the production of high-purity nanoplates with uniform morphology and satisfactory productivity, providing critical parameters for subsequent scale-up experiments.

#### 3.4.3. Size Control via Seed Concentration

**Figure 5:**
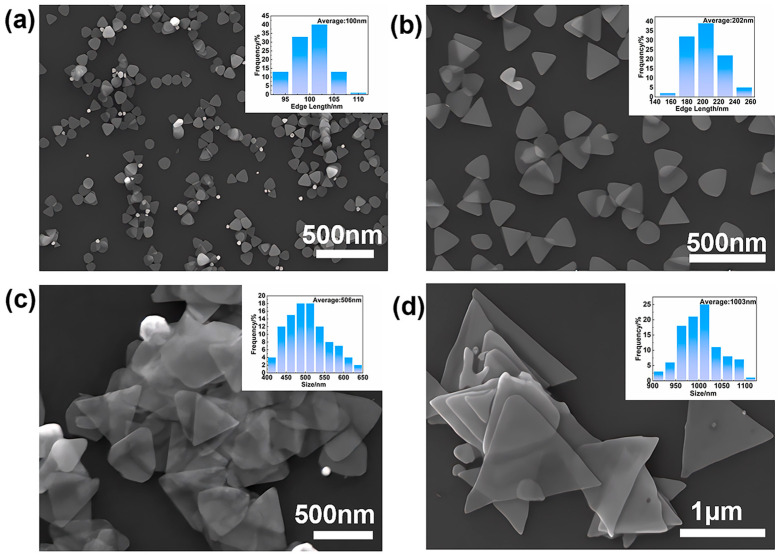
SEM images of silver nanoplates with tunable sizes achieved by adjusting seed solution volume. The images correspond to seed volumes of (**a**) 250 mL, (**b**) 150 mL, (**c**) 100 mL, and (**d**) 50 mL, respectively. The average sizes of the resulting triangular silver nanoplates are measured to be 100 nm, 202 nm, 506 nm, and 1003 nm for (a–d), respectively. Insets show the corresponding size distributions.

Figure 5 presents SEM images of silver nanoplates obtained through different amounts of Ag seeds. The characterization clearly demonstrated by precisely tuning the seed concentration; we successfully synthesized silver nanoplates with tunable edge lengths ranging from 100 nm to 1 μm. It is notable that achieving larger target sizes necessitates appropriately extending the reaction time to allow for sufficient growth.

### 3.5. Electrical Conductivity of Silver Nanoplate Pastes

**Figure 6:**
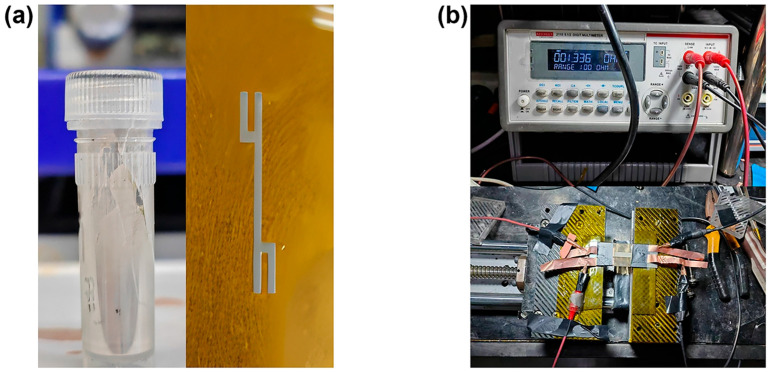
(**a**) Conductive silver paste and screen-printed patterns. Images show the synthesized silver nanoplates incorporated into conductive paste, and the resulting patterns screen-printed onto a PET film. (**b**) Setup for resistance measurement. A photograph illustrating the method used to measure the resistance values of the printed patterns.

Silver nanoplates, given their unique morphology, are highly valued as conductive fillers in applications such as conductive pastes. In this study, we incorporated the continuously flow-synthesized silver nanoplates into conductive pastes at specific doping ratios (51%) and evaluated their electrical properties (Figure 6). The mean thickness of the printed shape was about 3.67 μm; the length and width were 15 mm and 1.5 mm, respectively.

Table 1 presents the average resistivity of pastes prepared using silver nanoplates of three different sizes as conductive fillers. The experimental results clearly demonstrate a significant correlation between the resistivity of the conductive pastes and the size of the silver nanoplates. When using nanoplates with an average diameter of 1003 nm as conductive fillers, the resulting paste exhibited optimal conductivity with an impressively low average resistivity of 1.97 × 10^−7^ Ω·m. The paste prepared with nanoplates with an average diameter of 506 nm showed a slightly higher average resistivity (2.87 × 10^−7^ Ω·m). In contrast, the paste containing nanoplates with an average diameter of 202 nm displayed significantly poorer conductivity, with an average resistivity of 9.3 × 10^−7^ Ω·m.

In conductive paste systems, the quality of the conductive network formation is the primary determinant of electrical performance. The inherent advantage of silver nanoplates lies in their unique geometry, which facilitates face-to-face and line-to-line contacts. These types of contacts provide superior conductivity compared to conventional point contacts. Specifically, larger nanoplates offer a greater contact area and higher contact probability, thereby forming more continuous electron transport channels and significantly enhancing overall conductivity. Conversely, when the size of nanoplates decreases to a certain extent, the proportion of less efficient point contacts increases while face contacts decrease, leading to discontinuous conductive networks and consequently much higher resistivity.

It is noteworthy that while the electrical conductivity of silver nanoplates with an average diameter of 506 nm is slightly inferior to that of nanoplates with an average diameter of 1003 nm, the smaller one offers other advantages, including the potential for higher printing resolution and superior low-temperature sintering activity, which can lead to better overall performance in certain applications. Moreover, considering that larger sizes require longer processing times for preparation, from an industrial production perspective, selecting 500 nm as the target size level strikes an optimal balance. It ensures good conductivity while significantly improving production efficiency, making it more suitable for large-scale applications.

### 3.6. Scaled-Up Production and Future Prospects

After systematically investigating the effects of temperature, flow rate, and seed volume on the morphology and size of silver nanoplates, we successfully established an optimized protocol suitable for the large-scale production of high-quality silver nanoplates. The optimized conditions include a constant reaction temperature of 5 °C, a controlled flow rate of 708 μL/min (resulting in a 20 min residence time in the ice-water bath), and a target nanoplate size of 500 nm.

**Figure 7:**
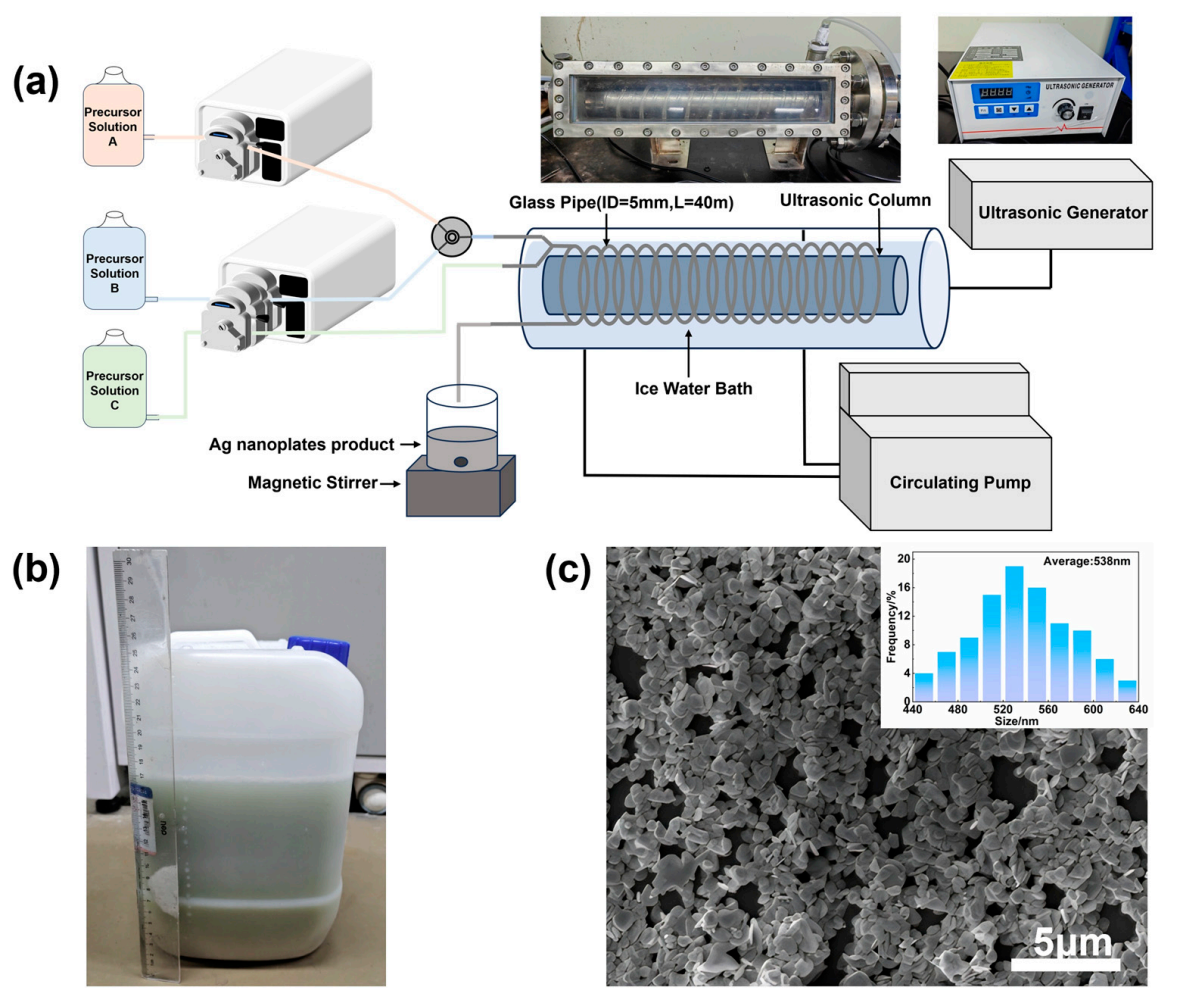
Mass production of silver nanoplates. (**a**) Schematic illustration of the scaled-up continuous-flow reactor equipment configuration. (**b**) Photograph of the silver nanoplate dispersion product collected in a large storage tank. (**c**) SEM image confirming the excellent morphological uniformity of silver nanoplates obtained from the scaled-up synthesis system and the inset shows the corresponding size distributions.

Building upon these optimized parameters, we designed and constructed a scalable synthesis system for large-scale production trials. As shown in Figure 7a, the setup utilizes two peristaltic pumps (one single-channel and one dual-channel) to accurately deliver the three reactant streams. These streams are thoroughly mixed through a static mixer before entering the reaction zone, which then passes through the ice-water bath ultrasonic reactor. The product dispersion is collected under continuous stirring to maintain homogeneity. In this scaled-up synthesis, the ultrasonic reaction zone incorporated 40 m of pipe with an inner diameter of 5 mm, achieving a throughput of 40 mL/min while critically maintaining the 20 min residence time. The entire device setup (Figure 7a) demonstrates a high level of integration, resembling industrial manufacturing processes.

Figure 7b shows a large storage tank containing the as-synthesized silver nanoplates dispersion, visually confirming the successful large-scale synthesis. Figure 7c further confirms that the silver nanoplates produced in this scaled-up synthesis possess good morphological uniformity, consistent with our laboratory-scale findings. Critically, this scaled-up system achieved a remarkable production capacity of 3.8 g/h of dried silver nanoplates with over 98% yield. This continuous flow synthesis approach enables daily production at the hectogram scale, as anticipated from our initial projections. Furthermore, the production capacity can be readily increased through the parallel multiplication of reaction channels, demonstrating the excellent potential of this method for industrial scale-up. This work represents a significant step towards the practical, high-volume manufacturing of high-quality silver nanoplates.

## 4. Conclusions

In this study, we successfully developed a continuous-flow reaction system for the scalable production of silver nanoplates. A key innovation lies in our integration of ultrasound-assisted technology, which effectively addresses common challenges in continuous-flow synthesis such as particle deposition and channel clogging. This enabled us to achieve highly uniform nanoplate morphology and precise size control.

Through a synergistic ultrasound-low temperature design, we established optimal growth conditions within a 5 mm inner diameter PTFE pipe, utilizing a 20 min residence time at 5 °C. Based on these optimized parameters, we designed and constructed a scaled-up production facility capable of achieving a remarkable daily output at the hectogram scale.

The as-prepared 500 nm silver nanoplates exhibited excellent electrical performance when incorporated into conductive pastes, demonstrating a remarkably low resistivity of 2.87 × 10^−7^ Ω·m. This work provides a practical and efficient solution for the large-scale production of high-quality silver nanoplates, holding strong potential to advance the industrialization of next-generation HJT solar cells and other applications reliant on silver nanomaterials. Furthermore, the versatility of this reactor system suggests it can be readily adapted for the scalable production of other nanomaterials through appropriate modifications.

## Figures and Tables

**Table 1:** The electrical properties of conductive silver paste composed with Ag nanoplates of different sizes after curing at 150 °C for 20 min.

Size (nm)	Resistance (Ω)	Resistivity Value (Ω·m)
1003 ± 100	0.54	1.97 × 10^−7^
506 ± 120	0.78	2.87 × 10^−7^
202 ± 40	2.53	9.3 × 10^−7^

## Data Availability

The data are available upon request from the corresponding author.
